# Increased risk of biliary infection after biliary stent placement in users of proton pump inhibitors

**DOI:** 10.1002/deo2.129

**Published:** 2022-05-22

**Authors:** Ryunosuke Hakuta, Yousuke Nakai, Hiroki Oyama, Kensaku Noguchi, Sachiko Kanai, Yusuke Nomura, Tatsunori Suzuki, Kazunaga Ishigaki, Kei Saito, Tomotaka Saito, Tsuyoshi Hamada, Naminatsu Takahara, Suguru Mizuno, Hirofumi Kogure, Kyoji Moriya, Mitsuhiro Fujishiro

**Affiliations:** ^1^ Department of Gastroenterology Graduate School of Medicine The University of Tokyo Tokyo Japan; ^2^ Department of Endoscopy and Endoscopic Surgery The University of Tokyo Tokyo Japan; ^3^ Department of Infection Control and Prevention Graduate School of Medicine The University of Tokyo Tokyo Japan

**Keywords:** cholangitis, cholecystitis, endoscopic retrograde cholangiopancreatography, proton pump inhibitors, stents

## Abstract

**Objectives:**

Proton pump inhibitors (PPIs) are widely prescribed medications for gastric acid‐induced diseases. Despite the effectiveness of PPIs, recent evidence suggested an increased risk of various bacterial infections in PPI users. The current study was conducted to evaluate the risk of biliary infection after endoscopic biliary stent placement in regular users of PPIs.

**Methods:**

Consecutive patients with a native papilla who underwent endoscopic retrograde cholangiopancreatography and stent placement for biliary stricture between January 2010 and August 2019 were included in this retrospective study. The cumulative incidences of biliary infection were compared between regular and non‐regular PPI users.

**Results:**

During the study period, 270 regular PPI users and 146 non‐regular PPI users were included in the analyses. Age, gender, and indication of endoscopic retrograde cholangiopancreatography were not different between the two groups. The incidences of biliary infection were 43% in regular PPI users and 36% in non‐regular PPI users but the time to biliary infection was significantly shorter in regular PPI users than in non‐regular users (28 vs. 87 days, *p* = 0.01). The cumulative incidence of biliary infection was significantly higher in regular PPI users compared with non‐regular users (*p* = 0.008). The multivariable Cox regression analysis also showed a significantly higher hazard ratio of biliary infection in regular PPI users (1.62 [95% confidence interval 1.16–2.26; *p* = 0.005]).

**Conclusions:**

Regular PPI use was associated with a higher risk of biliary infection after endoscopic biliary drainage. Inappropriate PPI use should be avoided.

## INTRODUCTION

Proton pump inhibitors (PPIs) are commonly prescribed medications for treating and preventing gastric acid‐induced diseases including gastroesophageal reflux disease or peptic ulcer disease.^1^ Due to their tolerable side effect, PPIs are often prescribed for a long period.^2^ Despite the promising effect of PPIs, recent studies suggested an increased risk of bacterial infection in PPI users including cholangitis.^3^, ^4^, ^5^, ^6^, ^7^ PPI use is supposed to decrease defense against pathogens through the suppression of gastric acid, and subsequently increases pathogenic Enterobacteriaceae.^8^

Endoscopic retrograde cholangiopancreatography (ERCP) and biliary stent placement are the standardized treatment for obstructive jaundice. Endoscopic stent placement across the biliary stricture recovers normal bile flow, but simultaneously implants gut bacteria into biliary systems. Therefore, patients after biliary stent placement predispose to a risk of biliary infection including cholangitis, cholecystitis, and liver abscess.^9^ We previously reported an increased risk of cholangitis with multi‐drug resistant bacteria in regular PPI users, particularly after multiple ERCP sessions.^10^ Considering the reduced protective mechanism against bacterial infection,^11^, ^12^ we hypothesized that regular PPI users have an increased risk of biliary infection not only with multi‐drug resistant bacteria.

Therefore, we conducted the current study to evaluate the association between regular PPI use and the incidence of biliary infection after endoscopic stent placement.

## METHODS

### Study design and population

The current study was a single‐center retrospective cohort study to evaluate the association between PPI use and biliary infection after biliary stent placement. We included consecutive patients who underwent ERCP and initial biliary drainage for biliary stricture during the study period using our prospectively maintained ERCP database. The exclusion criteria were as follows: 1) patients with a previous history of ERCP, 2) patients with Billroth‐II or Roux‐en‐Y reconstruction, and 3) patients with Billroth‐I reconstruction. The primary endpoint of this study was the cumulative incidence of biliary infection after endoscopic biliary stent placement. Secondary outcomes included time to biliary infection, specific details of biliary infection, and bile culture at the time of biliary infection.

This study was conducted according to the guidelines in the Helsinki Declaration and was approved by the ethics committee of the University of Tokyo. Written informed consent for ERCP was obtained from each patient before the procedure, and consent for the use of data for research was obtained on an opt‐out basis.

### Ascertainment of PPIs and definition of outcomes

Medications that were administered for each patient were ascertained using the electronic medical records at the hospital. Patients who took any PPI medication at the first session of ERCP during the study period were categorized as regular PPI users. Omeprazole, lansoprazole, rabeprazole, esomeprazole, and vonoprazan were categorized as PPI in this study.

Biliary infection was defined as cholangitis, cholecystitis, or liver abscess after biliary stent placement. Cholangitis which did not require biliary drainage was not included in biliary infection in this study. The diagnosis and severity of cholangitis and cholecystitis were defined in accordance with Tokyo Guidelines 2018.^13^ Bile sample was obtained for culture through a catheter during ERCP or endoscopic nasobiliary drainage (ENBD) at the time of biliary infection. Time to biliary infection was counted from biliary stent placement until biliary infection. For patients who underwent ENBD in the first session of ERCP, time to biliary infection was counted from the day of conversion to endoscopic biliary stenting from ENBD.

### Endoscopic procedures and follow‐up strategy after biliary stent placement

All endoscopic procedures were performed on an inpatient basis. Second‐generation cephalosporin was administered for prophylaxis before ERCP until the day after the index procedure. A side‐viewing duodenoscope (JF‐260V or TJF‐260V; Olympus, Tokyo, Japan, or ED‐580T; Fujifilm, Tokyo, Japan) was inserted under moderate sedation using hydrochloride pethidine with diazepam or midazolam. Plastic or a metal stent was placed across the biliary stricture. 7‐F or 8.5‐F plastic stents or metallic stents with a diameter of 8 or 10 mm and a length of 6 or 8 cm were used. For patients with hilar biliary stricture, a biliary stent was placed above the papilla. ENBD was selected for patients with concomitant cholangitis at the time of ERCP or with hilar cholangiocarcinoma. ENBD was converted to endoscopic biliary stenting after improvement of cholangitis or obstructive jaundice. Biliary stent replacement without biliary infection was scheduled in 3–6 months for patients with a benign biliary stricture.

Every patient who underwent endoscopic biliary stent placement was followed on an outpatient basis at least once a month. If patients had a fever, jaundice, or elevated liver enzyme, computed tomography was performed to diagnose biliary infection after biliary stent placement.

### Statistical analysis

Categorical variables were compared using the chi‐square test or Fisher's exact test, as appropriate. Continuous variables were compared using the Wilcoxon rank‐sum test.

Cumulative incidences of biliary infection after endoscopic biliary stent placement were estimated using the Kaplan‐Meier method and compared between regular and non‐regular PPI users using the log‐rank test. A scheduled biliary stent replacement without biliary infection was dealt with as a censor in the analyses. A Multivariable Cox regression model was used to examine the association between regular PPI use and biliary infection. Univariable analyses were performed to identify potential confounding factors. The multivariable model included regular use of PPI (regular use vs. non‐regular use) and variables with a *p‐*value of <0.15 in the univariable model. Subgroup analysis by the location of stent placement was also added.

For all analyses, a two‐sided *p‐*value <0.05 was used to denote statistical significance. All statistical analyses were performed using the EZR software (Saitama Medical Center, Jichi Medical University, Saitama, Japan), which is a graphical user interface for the R software (Vienna, Austria, version 3.4.1).^14^

## RESULTS

**FIGURE 1:**
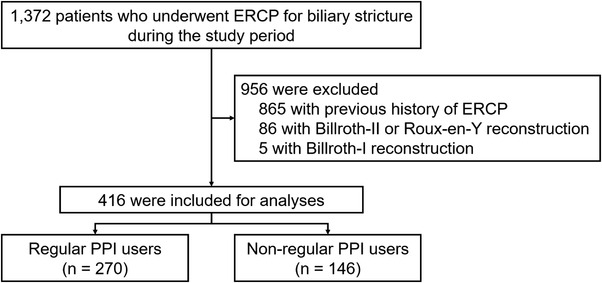
Flowchart of selection of patients who underwent endoscopic biliary stent placement for biliary stricture. ERCP, endoscopic retrograde cholangiopancreatography; PPI, proton pump inhibitor.

A total of 1,372 patients underwent ERCP for biliary stricture between January 2010 and August 2019. After excluding 956 patients, 416 eligible patients (270 regular PPI users and 146 non‐regular PPI users) were included in the analyses (Figure 1).

Baseline characteristics are shown in Table 1. There were no significant differences in age and gender between the two groups. The rate of malignant biliary obstruction was 89% and 94%, and that of benign biliary stricture was 11% and 6.2% in the regular and non‐regular PPI users, respectively. The rate of hilar stricture was higher in the regular PPI users (45% vs. 34%; *p* = 0.02). As for the underlying malignancy, pancreatic cancer was more frequent in non‐regular PPI users (32% vs. 45%, *p* = 0.01). The other baseline characteristics, including concomitant cholangitis, chemotherapy, and diabetes mellitus, were not different between the groups.

**TABLE 1:** Baseline characteristics of regular and non‐regular users of proton pump inhibitors who underwent endoscopic biliary stent placement

	PPI use		
Characteristic^†^	Regular users (*n* = 270)	Non‐regular users (*n* = 146)	*p‐*value
Gender			0.67
Male	169 (63%)	88 (60%)	
Female	101 (37%)	58 (39%)	
Age, years	70 (60–77)	70 (62–70)	0.40
Indication for ERCP			0.11
Malignant biliary obstruction	240 (89%)	137 (94%)	
Benign biliary stricture	30 (11%)	9 (6.2%)	
Concomitant cholangitis	17 (6.3%)	8 (5.5%)	0.83
Location of stricture			0.02
Hilar	122 (45%)	49 (34%)	
Distal	148 (55%)	97 (66%)	
Underlying malignancy			
Biliary tract cancer	99 (37%)	44 (30%)	0.20
Pancreatic cancer	86 (32%)	65 (45%)	0.01
Other malignancy	55 (20%)	28 (19%)	0.80
Chemotherapy	156 (58%)	83 (57%)	0.92
Aspirin use	33 (12%)	10 (6.8%)	0.09
Steroid use^‡^	75 (28%)	29 (20%)	0.08
Immunosuppressive agents use	34 (13%)	2 (1.4%)	<0.001
Diabetes mellitus	73 (27%)	29 (20%)	0.12

^†^ Data are expressed as the number (percentage) of patients within a given group or as the median (interquartile range).

^‡^ Steroid use included patients who used steroids as antiemetics for chemotherapy.

ERCP, endoscopic retrograde cholangiopancreatography; PPI, proton pump inhibitor.

**FIGURE 2:**
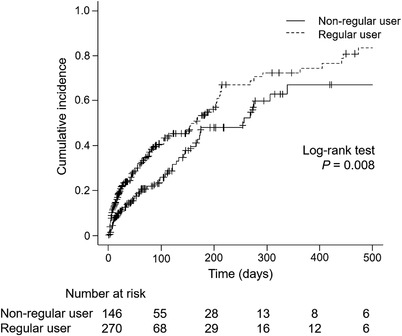
Cumulative incidences of biliary infection after endoscopic stent placement using Kaplan‐Meier method in regular and non‐regular users of proton pump inhibitors.

**FIGURE 3:**
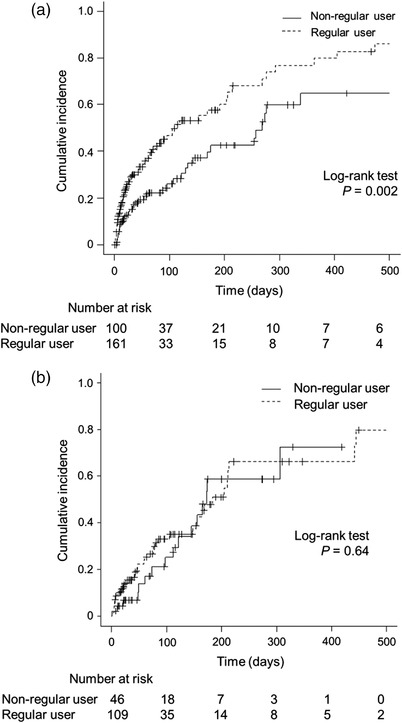
Cumulative incidences of biliary infection after endoscopic stent placement in regular and non‐regular users of proton pump inhibitors by the location of stent placement. Kaplan‐Meier curves in patients with (a) stent placement across the papilla, and (b) above the papilla.

Table 2 shows the details of endoscopic procedures. Half of the patients underwent metallic stent placement (47% in the regular PPI users and 49% in the non‐regular PPI users, respectively; *p* = 0.61). Cumulative incidences of biliary infections were significantly higher in regular PPI users than in non‐regular PPI users (*p* = 0.008; Figure 2). The incidences of biliary infection were 43% in regular PPI users and 36% in non‐regular PPI users (Table 3). Among 115 regular PPI users who developed a biliary infection, 102 patients (89%) continued PPI at the time of biliary infection. Time to biliary infection was significantly shorter in the regular PPI users (28 vs. 87 days, respectively; *p* = 0.01), during a median follow‐up period of 34 days in the regular PPI users and 63 days in the non‐regular PPI users, respectively. Cholangitis was the majority of biliary infections in both groups, and the severities of cholangitis were not different between the two groups. The multivariable‐adjusted hazard ratio of biliary infection was 1.62 (95% confidence interval 1.16–2.26, *p* = 0.005) in regular PPI users compared with non‐regular PPI users (Table 4). As for patients who developed cholangitis, the main cause of stent dysfunction was stone or sludge in both groups (19% and 16% in the regular and non‐regular PPI users, respectively), but the between‐group difference was not observed (*p* = 0.59; Table S1). Subgroup analyses by the location of biliary stricture are shown in Figure 3. In the subgroup of stent placement across the papilla, the cumulative incidence of biliary infection was significantly higher in regular PPI users than non‐regular users (*p* = 0.02), but the difference was not statistically significant in patients who underwent stent placement above the papilla.

**TABLE 2:** Endoscopic procedures in regular and non‐regular users of proton pump inhibitors

	PPI use		
Procedure^†^	Regular users (*n* = 270)	Non‐regular users (*n* = 146)	*p‐*value
Endoscopic biliary drainage			0.61
Plastic stent	144 (53%)	74 (51%)	
Metallic stent	126 (47%)	72 (49%)	
Type of Metallic stent			
Covered stent	82 (30%)^‡^	49 (34%)	0.51
Uncovered stent	46 (17%)	23 (16%)	0.78
Stent placement			0.09
Across the papilla	161 (60%)	100 (69%)	
Above the papilla	109 (40%)	46 (31%)	
Sphincteroplasty			
EST	122 (45%)	78 (53%)	0.12
EPBD	12 (4.4%)	3 (2.1%)	0.28

^†^ Data are expressed as the number (percentage) of patients within a given group.

^‡^ Two regular PPI users underwent both uncovered and covered metallic stent placement.

EPBD, endoscopic papillary balloon dilation; EST, endoscopic sphincterotomy; PPI, proton pump inhibitor.

**TABLE 3:** Incidences of biliary infection after endoscopic biliary stent placement in regular and non‐regular users of proton pump inhibitors

	PPI use		
Biliary infection^†^	Regular users (*n* = 270)	Non‐regular users (*n* = 146)	*p‐*value
Total	115 (43%)	53 (36%)	0.25
Time to biliary infection, median days	28	87	0.01
Cholangitis	112 (42%)	50 (34%)	0.17
Mild/moderate/severe	77/31/4 (29%/11%/1.5%)	33/15/2 (23%/10%/1.4%)	0.95
Time to cholangitis, median days	35	97	0.008
Cholecystitis	3 (1.1%)	3 (2.1%)	0.43
Time to cholecystitis, median days	7	10	0.51
Liver abscess	2 (0.7%)^‡^	0	0.54
Time to a liver abscess, median days	21	NA	
Follow up period, median days	34	63	0.003

^†^ Data are expressed as the number (percentage) of patients within a given group.

^‡^ Two regular PPI users developed both cholangitis and liver abscess.

PPI, proton pump inhibitor.

**TABLE 4:** Uni‐ and multivariable Cox regression analyses to assess the association between the use of proton pump inhibitors and biliary infection

			HR (95% CI) for BI			
Subgroup^†^	No.	BI, *n* (%)	Univariable	*p*‐value	Multivariable	*p‐*value
PPI						
Non‐regular users	146	53 (36%)	1 (referent)		1 (referent)	
Regular users	270	115 (43%)	1.56 (1.12–2.17)	0.008	1.62 (1.16–2.26)	0.005
Gender						
Female	159	61 (38%)	1 (referent)		1 (referent)	
Male	257	107 (42%)	1.26 (0.92–1.73)	0.14	1.20 (0.87–1.66)	0.27
Age						
<70 years	204	79 (39%)	1 (referent)			
≥70 years	212	89 (42%)	1.24 (0.91–1.68)	0.17		
Indication for ERCP						
BBS	39	8 (21%)	1 (referent)		1 (referent)	
MBO	377	160 (42%)	2.76 (1.35–5.62)	0.005	2.99 (0.96–9.38)	0.06
Location of stricture						
Distal	245	103 (42%)	1 (referent)			
Hilar	171	65 (38%)	0.96 (0.70–1.31)	0.79		
Concomitant cholangitis						
No	394	159 (41%)	1 (referent)			
Yes	25	9 (369%)	0.91 (0.47–1.79)	0.79		
Chemotherapy						
No	177	59 (33%)	1 (referent)		1 (referent)	
Yes	239	109 (46%)	1.41 (1.03–1.95)	0.03	1.45 (1.01–2.07)	0.04
Diabetes mellitus						
No	315	121 (39%)	1 (referent)			
Yes	102	47 (46%)	1.22 (0.87–1.71)	0.26		
Aspirin use						
No	373	146 (39%)	1 (referent)		1 (referent)	
Yes	43	22 (51%)	1.78 (1.13–2.79)	0.01	1.59 (1.00–2.54)	0.05
Steroid use						
No	312	123 (39%)	1 (referent)			
Yes	104	45 (43%)	0.97 (0.69–1.36)	0.84		
Immunosuppressive agents use						
No	380	160 (42%)	1 (referent)		1 (referent)	
Yes	36	8 (22%)	0.44 (0.22–0.89)	0.02	0.80 (0.26–2.46)	0.70
Endoscopic biliary drainage						
Plastic stent	218	80 (37%)	1 (referent)		1 (referent)	
Metallic stent	198	88 (44%)	0.74 (0.55–1.01)	0.06	0.54 (0.39–0.77)	<0.001
Stent placement						
Above the papilla	155	57 (37%)	1 (referent)			
Across the papilla	261	111 (43%)	1.26 (0.91–1.74)	0.16		
Sphincteroplasty						
No	201	72 (36%)	1 (referent)			
EST or EPBD	215	96 (45%)	1.14 (0.84–1.56)	0.39		

^†^ Use of proton pump inhibitors and variables with a *p‐*value <0.15 in the univariable model were entered into the multivariable model.

BBS, benign biliary stricture; BI, biliary infection; CI, confidence interval; EPBD, endoscopic papillary balloon dilation; ERCP, endoscopic retrograde cholangiopancreatography; EST, endoscopic sphincterotomy; HR, hazard ratio; MBO, malignant biliary obstruction; PPI, proton pump inhibitor.

The specific details of bile culture at the time of biliary infection in regular and non‐regular PPI users were not different (Table S2). The numbers of species identified from bile culture were neither different between the groups (median 2 vs. 2 in regular and non‐regular PPI users; *p* = 0.75). Among 149 non‐regular PPI users, eight patients used histamine‐2 antagonists and four (50%) developed a biliary infection after stent placement.

## DISCUSSION

The current study evaluated the association between regular PPI use and biliary infection after endoscopic biliary stent placement. The cumulative incidence of biliary infection was higher in regular PPI users compared with non‐regular users. In the multivariable Cox regression analyses, regular PPI use was also significantly associated with a higher hazard ratio of biliary infection. In addition, the median time to biliary infection was significantly shorter in regular PPI users than in non‐regular users.

Gastrointestinal microbiomes play an important role in the immune system against enteral bacterial infection,^11^ and their alteration contributes to increased morbidity and mortality of various enteric infections,^12^, ^15^ Among them, Clostridioides difficile colitis is a well‐known complicated disease after the prolonged use of antibiotic agents that decrease normal bacterial flora through the bactericidal effect.^3^ PPIs are also reported to increase Clostridioides difficile colitis and other bacterial infections including spontaneous bacterial peritonitis, and small intestinal bacterial overgrowth in addition to cholangitis.^4^, ^5^, ^7^, ^16^, ^17^ PPI use was reportedly to decrease gut microbiome diversity and increase Enterobacteriaceae.^8^, ^18^ Biliary systems originally maintained asepsis condition, but biliary intervention contaminates gastrointestinal bacteria into the biliary system.^19^ Considering the association between PPI use and increased pathogenic gut bacteria,^8^ regular PPI users are thought to be prone to biliary infection after the biliary intervention.

Most importantly, the current study showed the increased risk of biliary infection in regular PPI users after endoscopic biliary stent placement. Our previous retrospective cohort study showed a significantly higher rate of cholangitis with multi‐drug resistant bacteria in regular PPI users than in non‐regular users.^10^ The risk of cholangitis with multi‐drug resistant bacteria was further increased after multiple ERCP sessions. This study had heterogeneous inclusion criteria and patients without biliary stricture (e.g., bile duct stones) were included. Then, we focused on biliary infection after initial endoscopic stent placement for biliary stricture in the current study. A previous nationwide cohort study reported that the use of PPI increased the incidence of cholangitis, too.^7^ This study suggested a robust association between PPI use and an increased risk of cholangitis but did not show specific details of baseline characteristics or endoscopic procedures. In addition, one retrospective study evaluating the incidence of early biliary infection after percutaneous biliary stent placement showed an increased risk of biliary infection in PPI users.^20^ The result of the current study was also in line with these previous studies, and clearly showed a higher risk of biliary infection after endoscopic biliary stent placement in regular PPI users. Of note, the median time to biliary infection in regular PPI users was less than half compared with that of non‐regular PPI users. Inappropriate PPI use should be avoided to decrease the risk of biliary infection after biliary stent placement.

The alteration of gastrointestinal tract microbiome in PPI users was already reported.^18^ A previous cohort study showed an increase of genera *Enterococcus*, *Streptococcus*, *Staphylococcus*, and *Escherichia coli* in PPI users.^8^ In the current study, Enterococcus species tended to be higher in regular PPI users but the differences in specific species in bile culture were not statistically significant. Our relatively small sample size might be a reason for the null difference in the specific details of bile culture between the groups. Furthermore, contrary to our previous study in the consecutive ERCP cohort,^10^ the outcome of the current study was initial biliary infection after biliary stent placement. As our previous study suggested, the risk of multidrug‐resistant bacteria would increase as the number of ERCP sessions was more than three. Thus, the analysis of a limited cohort of the initial biliary stent placement with follow‐up to the first biliary infection episode may not allow the detection of differences in details of bacteria from bile culture.

Due to the retrospective nature of this study, some baseline characteristics were different between the groups. Among them, the rate of hilar biliary stricture and biliary stent placement above the papilla was higher in the regular PPI users. Contrary to hilar stricture, the subgroup analysis of patients with distal biliary stricture also showed a significantly higher cumulative incidence of biliary infection in regular PPI users. PPI use was reportedly associated with bacterial overgrowth in the duodenum including *Escherichia coli* and *Klebsiella spp*.^21^, ^22^ Considering potential duodenobiliary reflux after biliary stent placement across the papilla,^23^ it is plausible that the risk of biliary infection increases in regular PPI users, especially after biliary stent placement across the papilla. In addition, PPI use was suggested to increase the incidence of bile duct stones.^24^ Biliary sludge or bacterial biofilm occlude biliary stent, which subsequently develops cholangitis,^25^ but an association between these factors and PPI use needs further investigation.

The strength of our study is the use of a prospectively maintained large‐scale ERCP database. Using our database, we enabled to evaluate various potential confounders including the location of biliary stricture, concomitant chemotherapy, or diabetes mellitus. We have some limitations in this study. At first, our single‐center retrospective study design is the major limitation. Due to the retrospective nature, some baseline characteristics and endoscopic procedures were different between the two groups. A further randomized control trial is needed to clarify the association between PPI use and increased biliary infection. In the second, the exposure period of PPI was not available from our database. A previous study suggested an association between the risk of cholangitis and the PPI exposure period.^7^ Most patients were referred for ERCP procedures and PPIs were prescribed from other hospitals in our study cohort and we could not obtain these data. In the third, the follow‐up period was longer in the non‐regular PPI users compared with the regular PPI users. However, the main cause of this difference was derived from the significantly shorter time to biliary infection after biliary stent placement in the regular PPI users. Finally, Helicobacter pylori are known to be associated with low‐acid status and altered bacterial flora in stomach^26^ which potentially have an influence on biliary tract infection. However, data on Helicobacter pylori status were not available in our study cohort.

In conclusion, regular PPI users have a higher risk of biliary infection after endoscopic biliary stent placement compared with non‐regular users, and inappropriate use of PPI should be avoided.

## CONFLICT OF INTEREST

Mitsuhiro Fujishiro received lecture honoraria from EA pharma, Takeda pharmaceuticals, Daiichi Sankyo Co., AstraZeneca Co., Olympus Co., and Fujifilm Co., and a research grant from Olympus Co, HOYA Pentax Co., Fujifilm Co., EA pharma, and Takeda pharmaceuticals, and scholarship grants from EA pharma, and Takeda Pharmaceuticals outside the submitted work.

Yousuke Nakai received lecture honoraria from Olympus Co. and a research grant from HOYA Pentax Co., Fujifilm Co., and Boston Scientific Japan, and scholarship grants from Boston Scientific Japan, and Gadelius medical outside the submitted work. 

## Supporting information




**Supplementary Table 1**. Cause of biliary stent dysfunction at the time of cholangitis in regular and non‐regular users of proton pump inhibitors.
**Supplementary Table 2**. Details of bile culture at the time of biliary infection after endoscopic biliary stent placement in regular and non‐regular users of proton pump inhibitors.
