# Supplementary material for: Increased risk of biliary infection after biliary stent placement in users of proton pump inhibitors
Source: DEN Open. 2022 May 22;3(1):e129. doi: 10.1002/deo2.129 (PMC9307719; doi:10.1002/deo2.129)
Supplement: Supplementary file 1 — Supplementary Table 1. Cause of biliary stent dysfunction at the time of cholangitis in regular and non‐regular users of proton pump inhibitors. Supplementary Table 2. Details of bile culture at the time of biliary infection after endoscopic biliary stent placement in regular and non‐regular users of proton pump inhibitors. [file DEO2-3-e129-s001.docx]

**Supplementary Table 1.** Cause of biliary stent dysfunction at the time of cholangitis in regular and non-regular users of proton pump inhibitors.

|  | PPI use | |  |
| --- | --- | --- | --- |
| **Cause of dysfunction**† | Regular users  (n = 270) | Non-regular users  (n = 146) | *P* value |
| Stone or sludge | 51 (19%) | 24 (16%) | 0.59 |
| Ingrowth or overgrowth | 15 (5.6%) | 7 (4.8%) | 0.82 |
| Non-occlusive cholangitis | 11 (4.1%) | 5 (3.4%) | 0.99 |
| Stent migration | 10 (3.7%) | 8 (5.5%) | 0.45 |
| Other | 25 (9.3%) | 6 (4.1%) | 0.08 |

† Data are expressed as number (percentage) of patients within a given group.

PPI, proton pump inhibitor.

**Supplementary Table 2.** Details of bile culture at the time of biliary infection after endoscopic biliary stent placement in regular and non-regular users of proton pump inhibitors.

|  | PPI use | |  |
| --- | --- | --- | --- |
| **Bile culture**† | Regular users‡  (n = 87) | Non-regular users‡  (n = 44) | *P* value |
| Aerobic bacteria |  |  |  |
| *Escherichia coli* | 15 (17%) | 9 (21%) | 0.64 |
| *Klebsiella pneumoniae* | 16 (18%) | 12 (27%) | 0.26 |
| *Klebsiella oxytoca* | 10 (12%) | 8 (18%) | 0.30 |
| *Klebsiella ornithinolytica* | 1 (1.1%) | 0 | 0.99 |
| *Klebsiella aerogenes* | 7 (8.0%) | 2 (4.5%) | 0.72 |
| *Pseudonomas aeruginosa* | 10 (12%) | 5 (11%) | 0.99 |
| *Enterobacter cloacae* | 18 (21%) | 9 (20%) | 0.99 |
| *Enterobacter* sp. | 1 (1.1%) | 2 (4.5%) | 0.26 |
| *Enterococcus faecalis* | 26 (30%) | 8 (18%) | 0.21 |
| *Enterococcus faecium* | 15 (17%) | 6 (14%) | 0.80 |
| *Enterococcus casseliflavus* | 5 (5.7%) | 3 (6.8%) | 0.99 |
| *Enterococcus raffinosus* | 2 (2.3%) | 1 (2.3%) | 0.99 |
| *Acinetobacter* sp. | 2 (2.3%) | 0 | 0.55 |
| *Citrobacter* sp. | 9 (10%) | 5 (11%) | 0.99 |
| *Aeromonas* sp. | 6 (6.9%) | 3 (6.8%) | 0.99 |
| *Proteus mirabilis* | 1 (1.1%) | 0 | 0.99 |
| *Proteus vulgaris* | 1 (1.1%) | 0 | 0.99 |
| *Serratia marcescens* | 0 | 1 (2.3%) | 0.34 |
| *Staphylococcus aureus* | 1 (1.1%) | 2 (4.5%) | 0.26 |
| *Staphylococcus epidermidis* | 4 (4.6%) | 0 | 0.30 |
| *Staphylococcus warneri* | 1 (1.1%) | 0 | 0.99 |
| *Streptococcus anginosus* | 4 (4.6%) | 5 (11%) | 0.16 |
| *Streptococcus intermedius* | 1 (1.1%) | 0 | 0.99 |
| *Streptococcus salivarius* | 1 (1.1%) | 0 | 0.99 |
| Anaerobic bacteria |  |  |  |
| *Clostridium perfringens* | 2 (2.3%) | 2 (4.5%) | 0.60 |
| *Bacteroides ovatus* | 1 (1.1%) | 0 | 0.99 |
| *Lactobacillus* sp. | 1 (1.1%) | 0 | 0.99 |
|  |  |  |  |
| Negative bile culture | 4 (4.6%) | 5 (11%) | 0.16 |
| Positive findings |  |  |  |
| Number of species identified |  |  | 0.75 |
| 1 | 22 (25%) | 11 (25%) |  |
| 2 | 33 (38%) | 13 (30%) |  |
| ≥3 | 28 (31%) | 15 (34%) |  |

† Data are expressed as number (percentage) of patients within a given group.

‡ Twenty-nine regular users and nine non-regular users of PPI did not evaluate bile culture at the time of biliary infection.

PPI, proton pump inhibitor.
